# Serum ferritin as an independent biomarker of subclinical synovitis in rheumatoid arthritis patients in clinical remission or low disease activity

**DOI:** 10.3389/fmed.2026.1921362

**Published:** 2026-09-14

**Authors:** Duygu Kurtulus, Adem Kucuk

**Affiliations:** 1 Department of Physical Medicine and Rehabilitation, Faculty of Medicine, Uskudar University, Memorial Ataşehir Hospital, Istanbul, Türkiye; 2 Department of Internal Medicine, Division of Rheumatology, Faculty of Medicine, Necmettin Erbakan University, Konya, Türkiye

**Keywords:** ferritin, Power Doppler, rheumatoid arthritis, synovitis, ultrasonography

## Abstract

**Background:**

Residual synovial inflammation may persist in patients with rheumatoid arthritis (RA) despite clinical remission or low disease activity. Conventional inflammatory markers such as C-reactive protein (CRP) and erythrocyte sedimentation rate (ESR) may not adequately reflect subclinical synovitis detected by musculoskeletal ultrasound. Ferritin, an acute-phase reactant involved in inflammatory signaling, may represent a complementary biomarker of ongoing synovial inflammation.

**Objective:**

To evaluate the association between serum ferritin levels and Power Doppler (PD)-detected synovitis in patients with RA who were in clinical remission or low disease activity.

**Methods:**

In this prospective, single-center, cross-sectional study, 188 individuals were enrolled, including 132 patients with RA receiving either conventional synthetic disease-modifying antirheumatic drugs or adalimumab therapy and 56 healthy controls. All patients with RA fulfilled the 2010 ACR/EULAR classification criteria and had DAS28-ESR scores <3.2. Musculoskeletal ultrasound was performed in all patients with RA, and PD positivity was defined as a patient-level PD score of ≥1. Ferritin was analyzed as both a continuous and a categorical variable. To account for potential data separation and model overfitting, Firth's penalized likelihood multivariable logistic regression models were constructed, including adjustments for baseline iron status (serum iron and hemoglobin). Receiver operating characteristic (ROC) analysis was used to assess diagnostic performance, validated internally via 1,000 bootstrap resamples, and compared against CRP and ESR using DeLong tests.

**Results:**

PD positivity was identified in 40 of 132 patients with RA (30.3%). Ferritin levels were significantly higher in PD-positive than in PD-negative patients (median 117.0 vs. 17.0 ng/mL, *p* < 0.001), whereas CRP (*p* = 0.872) and ESR (*p* = 0.974) did not differ. In univariable Firth logistic regression, each 10 ng/mL increase in ferritin was associated with higher odds of PD positivity (Firth OR 2.49, 95% CI 1.70–3.66; *p* < 0.001). In the multivariable model adjusted for age, sex, treatment group, CRP, and ESR, ferritin remained independently associated with PD positivity (adjusted Firth OR 2.51 per 10 ng/mL, 95% CI 1.66–3.79; *p* < 0.001), and this independence persisted after further adjustment for serum iron and hemoglobin (adjusted Firth OR 2.14, 95% CI 1.51–3.05; *p* < 0.001). ROC analysis demonstrated excellent discriminatory performance for ferritin, with an optimism-corrected AUC of 0.985 (bootstrap 95% CI 0.968–0.997), which was significantly superior to CRP (AUC 0.509, DeLong *p* < 0.001) and ESR (AUC 0.498, DeLong *p* < 0.001). An exploratory ferritin threshold of 70 ng/mL yielded 95.0% sensitivity (bootstrap 95% CI 86.7–100.0%), 94.6% specificity (bootstrap 95% CI 89.1–98.9%), 88.4% positive predictive value (bootstrap 95% CI 77.8–97.4%), and 97.8% negative predictive value (bootstrap 95% CI 94.2–100.0%).

**Conclusion:**

Serum ferritin was independently associated with ultrasound-detected synovial inflammation in patients with RA who were in clinical remission or low disease activity. In this cohort, ferritin showed higher discriminatory performance than CRP and ESR, supporting its potential role as a complementary biomarker of residual synovial inflammation. However, the proposed cutoff of 70 ng/mL must be regarded as exploratory, and independent external validation in longitudinal prospective cohorts is required before clinical implementation.

## Introduction

Rheumatoid arthritis (RA) is a chronic autoimmune disease characterized by persistent synovial inflammation, progressive joint destruction, and systemic manifestations (1). The primary goal of RA management is to achieve and maintain remission or low disease activity, commonly assessed using composite indices such as the Disease Activity Score in 28 joints (DAS28) together with acute-phase reactants including C-reactive protein (CRP) and erythrocyte sedimentation rate (ESR) (1, 2). However, accumulating evidence suggests that these conventional measures may not fully reflect ongoing synovial inflammation, particularly in patients classified as being in clinical remission (3).

Musculoskeletal ultrasound, especially Power Doppler (PD) imaging, has emerged as a sensitive modality for detecting subclinical synovitis (4). Several studies have demonstrated persistent synovial vascularization in patients meeting remission criteria, indicating a potential disconnect between clinical remission and underlying inflammatory activity (3, 5). Importantly, persistent PD-detected synovitis has been associated with an increased risk of disease flare and structural progression, even in patients considered clinically controlled (5). Identifying accessible biomarkers that reflect ultrasound-detected inflammation therefore remains an important unmet need in routine clinical practice (4).

Ferritin, traditionally recognized as an iron storage protein, is increasingly acknowledged as an acute-phase reactant involved in immune activation and inflammatory signaling (6). Beyond iron metabolism, ferritin has been linked to macrophage activation and cytokine-mediated pathways relevant to rheumatoid arthritis (6, 7). Crucially, while hyperferritinemia is a well-established feature of severe systemic inflammatory syndromes such as macrophage activation syndrome and Still's disease, its relationship with localized, low-grade synovial inflammation in RA patients maintained within the clinical remission or low disease activity spectrum remains poorly defined (6, 7). Whether serum ferritin captures ultrasound-detected residual synovitis independently of conventional acute-phase reactants and systemic iron status represents a key knowledge gap in current RA monitoring strategies.

The present study aimed to evaluate the association between serum ferritin levels and Power Doppler-detected synovitis in patients with RA who were in clinical remission or low disease activity. By examining ferritin in relation to ultrasound findings, conventional inflammatory markers, and iron parameters, we sought to determine whether ferritin may serve as a complementary biomarker of residual synovial inflammation.

## Materials and methods

### Study design and participant recruitment

This prospective, single-center, cross-sectional study was conducted at the Department of Rheumatology and Physical Medicine of a tertiary care hospital between December 2025 and February 2026. Participants were prospectively recruited and underwent clinical, laboratory, and ultrasonographic assessments at a single baseline time point. A total of 188 participants were enrolled and allocated to three groups: patients with rheumatoid arthritis (RA) receiving conventional synthetic disease-modifying antirheumatic drugs (csDMARDs) (*n* = 70), patients with RA receiving adalimumab therapy (*n* = 62), and healthy controls (*n* = 56). Consecutive sampling was utilized during the enrollment period; a participant flow diagram outlining screening, eligibility, and exclusions is provided in Supplementary Figure S1.

All patients with RA fulfilled the 2010 American College of Rheumatology/European League Against Rheumatism classification criteria for RA. Eligibility required clinical remission or low disease activity, defined as a Disease Activity Score in 28 joints using erythrocyte sedimentation rate (DAS28-ESR) <3.2 at the time of assessment. Patients had been receiving stable csDMARD or adalimumab therapy for at least 3 months prior to inclusion. Only patients treated with adalimumab were included in the biologic treatment cohort to minimize therapeutic heterogeneity driven by differing mechanisms of action among biologic agents.

Eligible participants were aged between 20 and 80 years and had a body mass index (BMI) between 18 and 25 kg/m2. The strict BMI selection criterion was intentionally implemented to eliminate non-alcoholic fatty liver disease, metabolic syndrome, and obesity-driven low-grade systemic inflammation as potential metabolic confounders of serum ferritin levels, although this design choice intentionally limits external generalizability to broader overweight RA populations.

Exclusion criteria were corticosteroid use within the previous 3 months, diabetes mellitus, hyperlipidemia, pregnancy, renal or hepatic dysfunction, active systemic infection, malignancy, recent iron supplementation, and anemia defined as hemoglobin <10 g/dL or laboratory-confirmed iron deficiency (serum iron <30 µg/dL and transferrin saturation <15%). Healthy controls were recruited from hospital personnel and community volunteers, screened via history and laboratory testing to confirm the absence of inflammatory, metabolic, or infectious conditions.

### Clinical and laboratory assessments

Demographic and clinical data were obtained from medical records and the institutional database. Laboratory evaluations included C-reactive protein (CRP), ESR, hemoglobin, serum iron, total iron binding capacity (TIBC), and serum ferritin, performed using standardized automated analyzers in the institutional central laboratory. Ferritin was examined both as a continuous variable (including log2 transformation) and categorically using an exploratory threshold of 70 ng/mL for subclinical synovitis. Institutional reference limits were CRP <0.5 mg/dL and ESR <20 mm/h.

### Musculoskeletal ultrasound protocol and blinding

Musculoskeletal ultrasound examinations were performed in all patients with RA using a portable high-resolution ultrasound system (Clarius™, Vancouver, Canada) equipped with a high-frequency linear superficial probe (7.5–15.0 MHz). To eliminate observer bias, all examinations were conducted by a single experienced physiatrist (10+ years in musculoskeletal ultrasound) who was fully blinded to patient laboratory findings (including ferritin levels), clinical joint counts, DAS28 scores, and treatment group allocation.

A standardized 22-joint ultrasound protocol was performed according to EULAR guidelines, systematically assessing bilateral wrists (radiocarpal, intercarpal, and distal radioulnar joints), 2nd to 5th metacarpophalangeal (MCP) joints, 2nd to 5th proximal interphalangeal (PIP) joints, as well as any additional clinically tender joints. Standardized machine settings were maintained across all scans to optimize low-flow blood detection: Power Doppler frequency was set to 7.5 MHz, pulse repetition frequency (PRF) to 750 Hz, low wall filter, and color gain adjusted just below the noise threshold to eliminate blooming artifacts. Transducer pressure was carefully minimized using an abundant layer of acoustic gel.

Synovial hypertrophy (gray-scale) and synovial vascularization (Power Doppler) were graded on a semi-quantitative 0–3 scale in accordance with OMERACT criteria. For patient-level primary analyses, Power Doppler (PD) positivity was defined as the presence of a semi-quantitative PD score ≥1 in at least one examined joint. Intra-observer reliability was evaluated in a random subgroup of 20 patients scanned twice on the same day (1 h interval), yielding excellent agreement for PD scoring (weighted kappa = 0.88).

### Statistical analysis

Sample size adequacy was evaluated based on the primary study objective of assessing the diagnostic performance of serum ferritin for Power Doppler (PD)-detected subclinical synovitis. Based on previous literature, the anticipated prevalence of subclinical synovitis in patients with RA in clinical remission or low disease activity (DAS28-ESR <3.2) was estimated at approximately 30% (3). Assuming a two-sided significance level (*α*) of 0.05, a statistical power (1−*β*) of 0.80, an allocation ratio of approximately 1:2.3 between PD-positive and PD-negative patients, and a target area under the receiver operating characteristic curve (AUC) of 0.75 compared with a null hypothesis AUC of 0.50, the estimated minimum sample size was 119 patients with RA (36 PD-positive and 83 PD-negative). The final analytic cohort comprised 132 patients with RA, including 40 PD-positive (30.3%) and 92 PD-negative (69.7%) patients, exceeding the estimated minimum sample size required for the ROC analysis.

All statistical analyses were performed using IBM SPSS Statistics (version 25.0) and Python version 3.10 (statsmodels, scipy, and scikit-learn packages). Non-normally distributed continuous variables (assessed via Shapiro–Wilk test) are presented as median (IQR), and categorical variables as frequencies (%). Three-group comparisons used Kruskal–Wallis or chi-square tests, while two-group RA comparisons used Mann–Whitney U, chi-square, or Fisher's exact tests.

To correct for data sparsity (quasi-complete separation) and low event-per-variable ratios, multivariable penalized likelihood logistic regression (Firth's method) was implemented. Two adjusted models were constructed: Model 1 included ferritin (per 10 ng/mL increase), CRP, ESR, age, sex, and treatment group; Model 2 further incorporated serum iron and hemoglobin to rule out iron-store confounding. To assess log-linearity, ferritin was also analyzed following log2 transformation. Sex-stratified analyses and a ferritin-by-sex interaction term were evaluated to control for baseline sex differences in ferritin.

Diagnostic performance of ferritin at an exploratory cutoff of 70 ng/mL was evaluated using ROC analysis, validated internally via 1,000 bootstrap resamples to report optimism-corrected AUC, sensitivity, specificity, PPV, and NPV with bootstrap 95% CIs. ROC curves of ferritin, CRP, and ESR were formally compared using DeLong tests. Two-sided *p*-values <0.05 were considered statistically significant.

## Results

The study included 188 individuals: 56 healthy controls and 132 patients with rheumatoid arthritis (RA). Among the patients with RA, 70 were receiving conventional synthetic disease-modifying antirheumatic drugs (csDMARDs) and 62 were receiving tumor necrosis factor inhibitor (TNFi) therapy. The primary analytic cohort therefore consisted of the 132 patients with RA. Power Doppler (PD) positivity, defined as a patient-level PD score of ≥1, was identified in 40 of 132 patients (30.3%).

The three groups were broadly comparable in age and sex distribution, with a predominance of female participants across all groups. In contrast, inflammatory markers differed across groups. Both C-reactive protein (CRP) and erythrocyte sedimentation rate (ESR) were higher in the RA groups than in healthy controls, while ferritin levels also varied significantly, with the highest median values observed in the csDMARD group (Table 1). Overall, although the patients were classified within the low disease activity spectrum, laboratory indices of inflammation remained numerically higher in RA than in controls.

**Table 1:** Baseline demographic and laboratory characteristics of the study groups.

Variable	Control (*n* = 56)	csDMARD (*n* = 70)	TNFi (*n* = 62)	*p* value
Age, years	49.0 (39.8–57.2)	51.0 (41.0–60.5)	53.0 (39.5–63.0)	0.554
Female sex, *n* (%)	44 (78.6)	52 (74.3)	50 (80.6)	0.669
Hemoglobin, g/dL	12.9 (11.6–13.9)	12.3 (11.8–13.3)	12.0 (11.1–13.4)	0.065
CRP, mg/dL	0.44 (0.30–0.68)	0.56 (0.36–0.87)	1.02 (0.43–1.96)	<0.001
ESR, mm/h	11.0 (10.8–14.0)	23.0 (12.8–39.0)	26.0 (17.3–39.2)	<0.001
Serum iron, µg/dL	58.0 (32.5–89.2)	48.0 (31.2–86.8)	55.0 (25.2–82.8)	0.535
Ferritin, ng/mL	16.0 (11.0–60.2)	45.0 (16.0–91.0)	22.0 (9.7–75.2)	0.039

Data are presented as median (IQR) or number (%). Continuous variables were compared using the Kruskal–Wallis test and categorical variables using the chi-square test.

A clear separation emerged when RA patients were stratified according to PD status. Ferritin levels were markedly higher in PD-positive patients than in PD-negative patients, with median values of 117.0 ng/mL (IQR 89.8–181.0) and 17.0 ng/mL (IQR 10.0–30.3), respectively (*p* < 0.001) (Table 2). By contrast, conventional acute-phase reactants showed no discriminatory value. Median CRP levels were 0.57 mg/dL (IQR 0.38–1.85) in PD-positive and 0.68 mg/dL (IQR 0.40–1.26) in PD-negative patients (*p* = 0.872), and ESR values were likewise similar (22.5 mm/h [IQR 12.0–44.0] vs. 23.0 mm/h [IQR 15.0–37.0], *p* = 0.974) (Table 2).

**Table 2:** Clinical and laboratory characteristics of RA patients according to PD status.

Variable	PD-negative (*n* = 92)	PD-positive (*n* = 40)	*p* value
Age, years	50.5 (40.0–59.2)	56.5 (47.0–64.8)	0.056
Female sex, *n* (%)	83 (90.2)	19 (47.5)	<0.001
csDMARD treatment, *n* (%)	45 (48.9)	25 (62.5)	0.212
TNFi treatment, *n* (%)	47 (51.1)	15 (37.5)	0.212
Hemoglobin, g/dL	12.0 (11.4–12.9)	13.2 (12.3–13.9)	<0.001
CRP, mg/dL	0.68 (0.40–1.26)	0.57 (0.38–1.85)	0.872
ESR, mm/h	23.0 (15.0–37.0)	22.5 (12.0–44.0)	0.974
Serum iron, µg/dL	46.0 (25.0–71.0)	72.0 (52.0–94.0)	<0.001
Ferritin, ng/mL	17.0 (10.0–30.3)	117.0 (89.8–181.0)	<0.001
Ferritin <70 ng/mL, *n* (%)	87 (94.6)	2 (5.0)	<0.001
Ferritin ≥70 ng/mL, *n* (%)	5 (5.4)	38 (95.0)	<0.001

PD positivity was defined as a patient-level PD score ≥1. Continuous variables were compared using the Mann–Whitney *U*-test and categorical variables using the chi-square test.

When ferritin was analyzed categorically using an exploratory threshold of 70 ng/mL, PD positivity was observed in 2 of 89 patients (2.2%) with ferritin <70 ng/mL, compared with 38 of 43 patients (88.4%) with ferritin ≥70 ng/mL (Table 2). To account for potential bias caused by data sparsity in contingency cells, Firth's penalized likelihood logistic regression was performed, yielding a penalised odds ratio (OR) of 245.00 (95% CI 51.79–1159.01, *p* < 0.001) for ferritin ≥70 ng/mL.

In univariable analysis, continuous ferritin was strongly associated with PD positivity (Firth OR 2.49 per 10 ng/mL increase, 95% CI 1.70–3.66, *p* < 0.001). To address potential model overfitting and data separation, multivariable logistic regression models were constructed using Firth's penalized likelihood estimation (Table 3). In Model 1 (adjusted for age, sex, treatment group, CRP, and ESR), ferritin remained independently associated with PD positivity (adjusted Firth OR 2.51 per 10 ng/mL increase, 95% CI 1.66–3.79, *p* < 0.001).

**Table 3:** Firth's penalized multivariable logistic regression analyses for PD positivity in RA patients.

Variable	Model 1 Adjusted Firth OR (95% CI)	*p* value	Model 2 (Iron & Hb Adjusted) Firth OR (95% CI)	*p* value
Ferritin (per 10 ng/mL increase)	2.51 (1.66–3.79)	<0.001	2.14 (1.51–3.05)	<0.001
CRP (mg/dL)	0.65 (0.35–1.19)	0.160	1.16 (0.85–1.58)	0.364
ESR (mm/h)	0.98 (0.93–1.03)	0.415	0.97 (0.92–1.02)	0.177
Age (years)	0.98 (0.91–1.05)	0.525	0.97 (0.90–1.04)	0.370
Female sex	0.42 (0.06–2.83)	0.370	0.96 (0.15–6.30)	0.966
TNFi treatment (vs. csDMARD)	0.81 (0.12–5.53)	0.833	0.89 (0.16–4.94)	0.898
Serum iron (µg/dL)	—	—	1.01 (0.99–1.04)	0.290
Hemoglobin (g/dL)	—	—	1.28 (0.64–2.55)	0.489

Model 1 includes ferritin, CRP, ESR, age, sex, and treatment group. Model 2 incorporates serum iron and hemoglobin as additional covariates.

Because PD-positive patients presented with significantly higher baseline hemoglobin and serum iron levels (Table 2), a sensitivity model (Model 2) was performed incorporating hemoglobin and serum iron to rule out iron-store confounding. Ferritin maintained its robust independent association with PD positivity (adjusted Firth OR 2.14 per 10 ng/mL increase, 95% CI 1.51–3.05, *p* < 0.001), whereas serum iron (OR 1.01, *p* = 0.290) and hemoglobin (OR 1.28, *p* = 0.489) were not independently associated with PD positivity. Furthermore, evaluating ferritin on a log2 scale to test linearity confirmed that each doubling of serum ferritin was associated with an adjusted Firth OR of 38.42 (95% CI 5.69–259.24, *p* = 0.0002).

Sex-stratified subgroup analyses demonstrated that the association between ferritin and PD positivity remained statistically significant in both females (*n* = 102; median ferritin 109.0 vs. 16.0 ng/mL, *p* < 0.001) and males (*n* = 30; median ferritin 154.0 vs. 47.0 ng/mL, *p* < 0.001).

Ferritin showed excellent discriminatory performance for PD-positive synovitis on receiver operating characteristic (ROC) analysis, with an apparent area under the curve (AUC) of 0.985 (95% CI 0.968–0.997). To correct for potential optimism bias, internal validation using 1,000 bootstrap resamples was conducted, yielding an optimism-corrected AUC of 0.985 (bootstrap 95% CI 0.968–0.997). Formal statistical comparison using DeLong tests demonstrated that the ROC curve for ferritin was significantly superior to both CRP (AUC 0.509, DeLong *z* = 8.25, *p* < 0.001) and ESR (AUC 0.498, DeLong *z* = 8.38, *p* < 0.001).

At the exploratory cutoff of 70 ng/mL derived via the Youden index, ferritin yielded an apparent sensitivity of 95.0% (bootstrap 95% CI 86.7–100.0%) and specificity of 94.6% (bootstrap 95% CI 89.1–98.9%). The positive predictive value was 88.4% (bootstrap 95% CI 77.8–97.4%) and negative predictive value was 97.8% (bootstrap 95% CI 94.2–100.0%) (Table 4).

**Table 4:** Diagnostic performance and bootstrap internal validation of ferritin for PD positivity.

Measure	Apparent Value	Bootstrap Internal Validation (95% CI)
AUC	0.985	0.985 (0.968–0.997)
Optimal cutoff, ng/mL	70	70 (Exploratory)
Sensitivity, %	95.0	95.0 (86.7–100.0)
Specificity, %	94.6	94.6 (89.1–98.9)
Positive predictive value, %	88.4	88.4 (77.8–97.4)
Negative predictive value, %	97.8	97.8 (94.2–100.0)

Bootstrap estimates and confidence intervals were calculated using 1,000 resamples.

Subgroup analyses according to treatment strategy confirmed that ferritin levels were significantly higher in PD-positive patients within both the csDMARD group (median 113.0 vs. 18.0 ng/mL, *p* < 0.001) and the TNFi group (median 174.0 vs. 15.0 ng/mL, *p* < 0.001), with no significant interaction between treatment type and ferritin (*p* = 0.833).

## Discussion

In this cross-sectional study of patients with rheumatoid arthritis (RA) who were in clinical remission or low disease activity, serum ferritin was strongly associated with Power Doppler (PD)-detected synovitis. Ferritin levels were substantially higher in PD-positive patients than in PD-negative patients, and this association remained significant after adjustment for age, sex, treatment group, C-reactive protein (CRP), erythrocyte sedimentation rate (ESR), serum iron, and hemoglobin. These findings suggest that higher ferritin levels may be associated with residual inflammatory activity not fully captured by conventional clinical and laboratory assessments. However, because of the cross-sectional design, these results demonstrate a cross-sectional association and must not be interpreted as establishing causation or predicting longitudinal outcomes such as disease flares or structural progression.

Although CRP and ESR remain standard components of RA disease assessment, their limitations are well recognized, particularly in patients with low-grade inflammation or those receiving biologic therapy (1, 2, 8, 9). Imaging studies have consistently shown that subclinical synovitis may persist despite apparent clinical control, and persistent PD activity has been associated with subsequent flare and structural progression (3, 5, 10–13, 16–19). In the present cohort, PD-positive synovitis was observed despite low conventional inflammatory markers, whereas ferritin levels showed a stronger cross-sectional association with PD status. This finding suggests that ferritin may capture aspects of inflammatory activity not adequately represented by standard acute-phase reactants; however, its clinical utility as a biomarker cannot be established from the present data.

Ferritin is increasingly understood as more than a marker of iron storage. In addition to its role in iron metabolism, it has been linked to macrophage activation and cytokine-mediated inflammatory responses relevant to RA pathophysiology (6, 7, 11, 14). Persistent low-grade synovial or tissue-level macrophage activity may contribute to elevated ferritin levels even in patients classified as being in clinical remission or low disease activity by composite indices such as DAS28. Although hyperferritinemia is classically recognized in severe inflammatory syndromes such as macrophage activation syndrome, the pathophysiology of these systemic hyperferritinemic conditions differs substantially from that of low-grade residual synovitis in RA. Therefore, the observed association between ferritin and PD-detected synovitis should be interpreted cautiously and does not establish ferritin as a specific marker of synovial inflammation.

Importantly, because PD-positive patients in our cohort exhibited higher baseline hemoglobin and serum iron levels, multivariable models were specifically adjusted for systemic iron parameters. The persistence of ferritin's independent association in these sensitivity analyses suggests that the observed relationship is not fully explained by differences in systemic iron status. Furthermore, penalised likelihood logistic regression (Firth's method) was used to reduce potential estimation bias arising from data sparsity in contingency cells, and the association remained consistent across linear and log-transformed ferritin scales.

ROC analyses demonstrated a high discriminatory ability of ferritin for PD-positive synovitis within this cohort, with significantly greater discrimination than CRP and ESR according to DeLong comparisons. Internal validation using 1,000 bootstrap resamples yielded an optimism-corrected AUC of 0.985. However, the magnitude of this AUC should be interpreted cautiously because it was derived from a highly selected study population with a restricted BMI range and exclusion of several relevant comorbidities. Moreover, bootstrap validation assesses internal optimism but does not substitute for external validation, and the cross-sectional design does not establish diagnostic or predictive performance in broader clinical populations. The threshold of 70 ng/mL should therefore not be regarded as an established clinical cutoff for subclinical synovitis; rather, it represents an exploratory threshold derived within this specific cohort. Independent external validation in larger, more heterogeneous populations is required before any clinical utility can be established.

From a clinical perspective, residual inflammation is especially relevant when treatment de-escalation, monitoring strategies, or flare risk are being evaluated (1, 5, 12, 13). Musculoskeletal ultrasound is sensitive for detecting subclinical synovitis, but its routine use may be limited by time, operator dependence, and availability (4, 10, 15). If the observed association is confirmed in prospective, externally validated studies, serum ferritin may warrant further investigation as a potential adjunct to clinical assessment when considering the need for additional imaging.

Several limitations should be acknowledged. First, the cross-sectional and single-center design precludes causal inference and limits temporal assessment. Second, the highly selected nature of the study population limits external generalizability. Enrollment was restricted to patients with a normal BMI range (18–25 kg/m²), and several relevant comorbidities that may influence ferritin levels were excluded to reduce potential confounding; consequently, the findings may not be generalizable to broader, more clinically heterogeneous RA populations. Third, RA-related clinical characteristics were not comprehensively characterized, including variables such as disease duration, serological status, and detailed treatment history, which may have influenced the observed association and limited adjustment for potential disease-related confounding. Fourth, a marked sex imbalance was observed between PD groups, although sex-stratified analyses showed consistent associations in both males and females. Fifth, ultrasound examinations were conducted by a single investigator, introducing potential operator dependence, although intra-reader reliability was excellent. Sixth, the proposed ferritin cutoff was derived internally without external validation. Finally, longitudinal clinical outcomes such as disease flare, radiographic progression, or therapeutic response were not evaluated; therefore, the prognostic value of serum ferritin remains to be established in prospective studies.

Despite these limitations, the present study demonstrates that serum ferritin was independently associated with PD-detected subclinical synovitis in RA patients with clinical remission or low disease activity. These findings support further investigation of the observed association in larger, more heterogeneous, and prospectively followed RA populations to determine whether ferritin has clinical value beyond conventional inflammatory markers.

## Conclusion

Serum ferritin was independently associated with ultrasound-detected synovial inflammation in patients with rheumatoid arthritis who were in clinical remission or low disease activity. In this selected cohort, ferritin showed greater discrimination between PD-positive and PD-negative patients than conventional inflammatory markers such as CRP and ESR**; however, this finding should be interpreted within the context of the restricted study population and lack of external validation.

These findings suggest that the observed association between ferritin and residual, low-grade synovial inflammation warrants further investigation, particularly as such inflammation may be not fully captured by standard clinical and laboratory assessments. However, these results support a cross-sectional association rather than a validated diagnostic test, and the proposed ferritin cutoff of 70 ng/mL must be regarded as purely exploratory. Clinical implementation or diagnostic screening should not be advocated until independent external validation is completed.

Prospective longitudinal studies in larger, more heterogeneous populations are needed to determine whether elevated serum ferritin levels predict subsequent disease flare, structural progression, or treatment outcomes.

## Data Availability

The raw data supporting the conclusions of this article will be made available by the authors, without undue reservation.
