# Supplementary material for: Serum ferritin as an independent biomarker of subclinical synovitis in rheumatoid arthritis patients in clinical remission or low disease activity
Source: Front Med (Lausanne). 2026 Sep 14;13:1921362. doi: 10.3389/fmed.2026.1921362 (PMC13616694; doi:10.3389/fmed.2026.1921362)
Supplement: Supplementary file 1 [file Table1.docx]

**Supplementary Figure S1. Flow diagram of participant recruitment and study group allocation.**
